# Inhibitory Effects of *Euphorbia tirucalli* Lineu (Euphorbiaceae) Diluted Latex on Human and Canine Melanoma Cells

**DOI:** 10.1155/2020/4093206

**Published:** 2020-07-14

**Authors:** Diego Pinha Alves da Paz, Márcia Kazumi Nagamine, Murilo Penteado Del Grande, João Vitor Pereira Leite, Flavia Mendonça Gonçalves Sobreira, Elfriede Marianne Bacchi, Maria Lucia Zaidan Dagli

**Affiliations:** ^1^School of Veterinary Medicine and Animal Science of the University of São Paulo, Laboratory of Experimental and Comparative Oncology, Department of Pathology, Av Prof. Dr. Orlando Marques de Paiva, 87 CEP 05508-900, São Paulo, SP, Brazil; ^2^School of Pharmaceutical Sciences of the University of São Paulo, Department of Pharmacy, Av. Professor Lineu Prestes, 580 CEP 05508-000, São Paulo, SP, Brazil

## Abstract

*Euphorbia tirucalli* Lineu (Euphorbiaceae) is a tropical and subtropical ornamental and toxic plant. *E. tirucalli* produces a latex that is commonly used to treat neoplasms. This study aimed to evaluate the effects of diluted *E. tirucalli* latex (DETL) on human (SK-MEL-28) and canine (CBMY) melanoma cells. SK-MEL-28 (3 × 10^3^ cells/well) and CBMY (6 × 10^3^ cells/well) were cultivated in 96-well plates. The cells were treated with 50 *μ*l/well of dilutions (1/2, 1/4, 1/8, 1/16, 1/32, 1/64, 1/128, 1/256, and 1/512) of a standard solution containing 1 mg/mL of the *E. tirucalli* latex (ETL) in DMEM. Control group cells received 50 *μ*l/well of DMEM. After 24, 48, and 72 h of treatment, cell viability was assessed by the MTT assay. There was a significant decrease in viability at 48 and 72 hours after treatment for human melanoma cells and at 24, 48, and 72 hours for canine cells, mainly in higher dilutions of ETL. Human melanoma cells presented a typical *U* shape curve, characteristic of hormesis. To our knowledge, this is the first study showing inhibitory effects of DETL on canine melanoma cells. Therefore, DETL is a potentially new antineoplastic drug.

## 1. Introduction

Melanomas in humans and canines are aggressive diseases, and the currently available treatments are generally inefficient [1]. Therefore, studies to identify other therapeutic possibilities, such as phytotherapy, have become relevant. *Euphorbia tirucalli* Lineu (Euphorbiaceae), commonly known as “aveloz,” is a succulent tropical and subtropical plant with many branches, which is usually commercialized as an ornamental plant [2]. Several reports indicate that *E. tirucalli* is toxic for dogs and cats [3] and fishes [4, 5] and has shown genotoxicity and cytotoxicity in human leukocytes [6], developmental toxicity in rats [7], and irritant and tumor-promoting activities in mice [8, 9]. The composition of *E. tirucalli* latex includes terpenes and sterols, some of which have already been isolated, such as taraxasterol and tirucallol, and euphol and alpha-euphorbol. The major components are water, tigliane, and ingenane [10, 11].

*E. tirucalli* produces a latex that is popularly used to treat tumors. In popular culture, *E. tirucalli* latex (ETL) is used to treat tumors at low concentrations, diluted in saline solution, and there are several reports of positive antineoplastic effects on experimental tumors or human cells. Extracts of *E. tirucalli* from three regions of Brazil presented antiproliferative activity against leukemia (HL-60), lymphoma (Daudi Burkitt's lymphoma cells), and B16/F10 melanoma cells [12]. High dilutions of *E. tirucalli* latex were effective in inhibiting the growth of human melanoma cells (MV3) [13] and HCT-116 colon cell lines [14], human breast cancer cells (MCF7), but not melan-A nonneoplastic melanocytic cell line [15].

Recently, it has been verified that euphol, a tetracyclic triterpene alcohol, the main constituent of *E. tirucalli* latex, exerted an inhibitory activity against human cancer cells in vitro and cytotoxicity against human glioblastoma cells in vitro and in vivo [16, 17]. *E. tirucalli* latex has also shown controversial effects. As stated above, it induced genotoxicity [6] and changes in antioxidant gene expression [18] in human leukocytes. In addition, it presented an angiogenic activity [19]; these activities could favor the promotion and progression of carcinogenesis. In addition, high dilutions have shown to interfere with the cell metabolism [15]. Therefore, it is important to conduct scientific studies to verify the popular cultural belief that the plant could treat cancer.

Dogs are considered good models for human cancers, because they spontaneously develop many types of neoplasms, which share similar characteristics with human counterparts [20]. Although diluted *E. tirucalli* latex (DETL) has been tested on human melanoma cells [13], to our knowledge, there are no studies to date verifying the therapeutic effects of *E. tirucalli* latex (ETL) or DETL on canine cells. Our hypothesis is that if inhibitory results are obtained, DETL could be used to treat canine oral melanomas.

This study aimed to evaluate the effects of *E. tirucalli* latex, diluted according to popular usage, on human and canine melanoma cells.

## 2. Materials and Methods

### 2.1. Cells

SK-MEL-28 cells (ATCC ®HTB-72™) were purchased from ATCC, then stored in the Laboratory of Experimental and Comparative Oncology of the School of Veterinary Medicine and Animal Science of the University of São Paulo (SVMAS-USP), and kept frozen at −80°C. Cells were thawed and cultured in flasks when necessary. A canine buccal melanoma cell line (CBMY) was created and characterized at the Laboratory of Experimental and Comparative Oncology of the SVMAS-USP. The CBMY cells originated from the oral melanoma of a 10-year-old male Yorkshire Terrier dog. Cells stained positively for Melan-A (Melan-A antibody (A103), Santa Cruz Biotechnology) and vimentin (Dako, Carpinteria, CA, USA) antibodies confirmed the tumor origin as melanoma (data not shown). Cells were cultured in DMEM medium supplemented with penicillin (50 IU/ml), streptomycin (50 mg/ml), and 10% fetal bovine serum, under standard incubator conditions (37°C, 5% CO_2_, humidified atmosphere).

### 2.2. *Euphorbia tirucalli* Lineu (Euphorbiaceae)

*E. tirucalli* Lineu (Euphorbiaceae) was obtained from a plant resource center in São Paulo, Brazil, and has been certified by the ECOCERT group (http://www.brazil.ecocert.com/index/). In addition, a sample of *E. tirucalli* pressed plant was obtained, dried in an oven, fixed on a standard size paperboard, accompanied by a label containing information about the plant and its collection site, and stored in the herbarium of the Department of Botany of the Institute of Biosciences of the University of São Paulo, Brazil (http://www.ib.usp.br/en/botany-welcome.html), under the code “PAZ 1 (SPF).” All ETL required for the experiments were collected directly from the stems of the plant in a sterilized glass beaker.

### 2.3. Dilutions

The dilution of *E. tirucalli* latex was standardized as 1 mg/mL of pure latex in DMEM, and this was denominated “standard solution.” For in vitro experiments, the standard solution was used, and the serial dilutions made with the culture medium were 1/2, 1/4, 1/8, 1/16, 1/32, 1/64, 1/128, 1/256, and 1/512. Immediately after the preparation, the diluted *E. tirucalli* latex solution was properly stored at 4°C.

### 2.4. In Vitro Experiment

On day 1, 3 × 10^3^ SK-MEL-28 melanoma cells/well (150 *μ*l/well) were plated onto 3 flat-bottomed 96-well plates for evaluation of the treatment after 24 h, 48 h, and 72 h. In a parallel experiment, 6 × 10^3^ CBMY cells/well (150 *μ*l/well) were plated in 3 flat-bottomed 96-well plates, also for the evaluation of the treatment after 24, 48, and 72 h. On the 2^nd^, 3^rd^, and 4^th^ days, SK-MEL-28 and CBMY melanoma cells were treated with 50 *μ*l/well serial dilutions (1/2 to 1/512) of the *E. tirucalli* latex standard solution. The dilutions were numbered as follows: 0 (*E. tirucalli* latex standard solution), 1 (1/2 dilution), 2 (1/4 dilution), 3 (1/8 dilution), 4 (1/16 dilution), 5 (1/32 dilution), 6 (1/64 dilution), 7 (1/128 dilution), 8 (1/256 dilution), and 9 (1/512 dilution). The control group (ct) received 50 *μ*l/well of DMEM as the vehicle. The two sets of plates (1, 2, and 3) with SK-MEL-28 and CBMY and cells were analyzed by colorimetric assay at 24, 48, and 72 h after treatment.

### 2.5. Evaluation of Cell Proliferation In Vitro: MTT Assay

To determine the effectiveness of the treatment, cell viability was assessed by the colorimetric test, MTT ([3- (4,5-dimethylthiazol-2yl)-2,5-diphenyl tetrazolium bromide]) [21]. After the treatments and required incubation time (24 h, 48 h, and 72 h), 20 *μ*l/well of MTT (5 mg/ml) was added to each plate 3 h before the end of the experiment. Cells treated with MTT were incubated in an oven and then centrifuged (4000 rpm/10 min). Subsequently, the medium was discarded and 100 *μ*l of DMSO was added. The optical density was set at 570 nm, read in a spectrophotometer (ELISA reader).

### 2.6. Statistical Analysis

Results were analyzed to verify normality and submitted to ANOVA for parametric data with Tukey's posttest. Results were considered significant when *p* < 0.05.

## 3. Results

**Figure 1:**
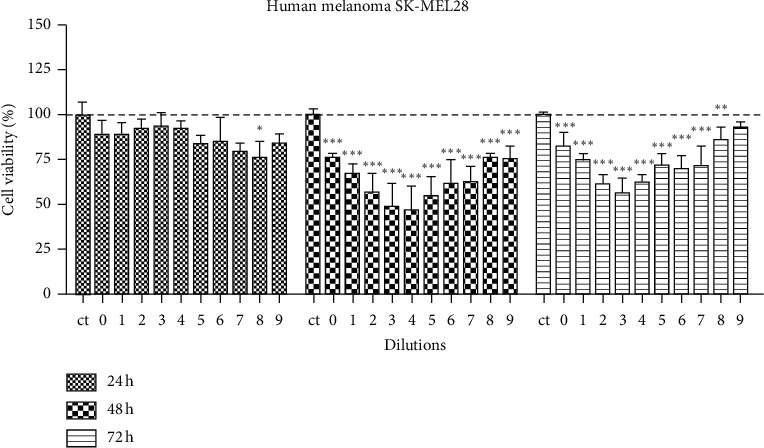
SK-MEL 28 human melanoma cell viability after treatment with different dilutions of *E. tirucalli* latex. The dilutions were numbered as follows: 0 (*E. tirucalli* latex standard solution–1 mg/mL), 1(1/2), 2(1/4), 3(1/8), 4(1/16), 5(1/32), 6(1/64), 7(1/128), 8(1/256), and 9(1/512). The control group (ct) received 50 *μ*l/well of saline solution. ^*∗*^*p* < 0.05,^*∗∗*^*p* < 0.01,^*∗∗∗*^*p* < 0.001.

Figure 1 shows the viability of SK-MEL 28 cells at 24, 48, and 72 h after treatment with 9 serial dilutions of *E. tirucalli* latex standard solution. Significant reductions (*p* < 0.05, *p* < 0.01, or *p* < 0.001) in cell viability at most dilutions were observed mainly at 48 and 72 h after treatment.

**Figure 2:**
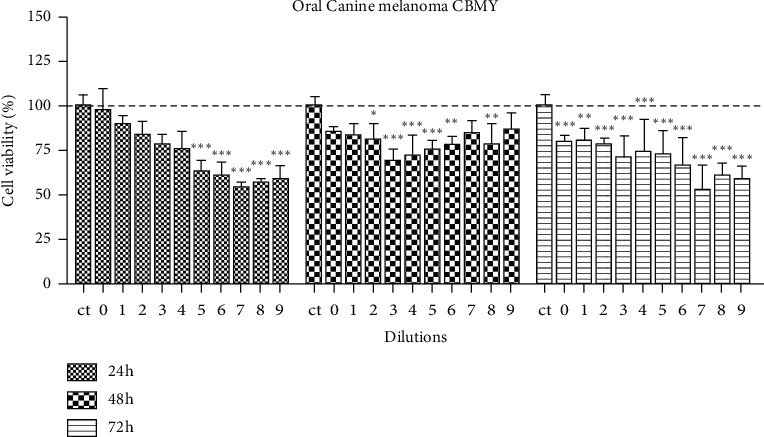
CBMY canine melanoma cell viability after treatment with different dilutions of *E. tirucalli* latex. The dilutions were numbered as follows: 0 (the *E. tirucalli* latex standard solution–1 mg/mL), 1(1/2), 2(1/4), 3(1/8), 4(1/16), 5(1/32), 6(1/64), 7(1/128), 8(1/256), and 9(1/512). The control group (ct) received 50 *μ*l/well of saline solution. ^*∗*^*p* < 0.05,^*∗∗*^*p* < 0.01,^*∗∗∗*^*p* < 0.001.

Figure 2 shows the viability of CBMY cells at 24, 48, and 72 h after treatment with 9 serial dilutions of the *E. tirucalli* latex standard solution. Significant reductions (*p* < 0.05, *p* < 0.01 or *p* < 0.001) in cell viability were observed at 24, 48, and 72 h after treatment, in different dilutions. However, the highest dilutions (from 5 to 9) showed notably higher inhibitory effects on cell viability at 24 and 72 h, in canine oral melanoma cell lines.

## 4. Discussion

*E. tirucalli* is an ornamental plant whose latex has shown toxic properties for some animal species, including human leukocytes [3–9], and has some procarcinogenic properties [6, 18–19]. On the other hand, it has been shown that some components of its latex can be used in the right dose to treat cancer [12–15]. This study aimed to verify if the commonly used dose of *E. tirucalli* latex is effective in reducing the viability of human and canine melanoma cells, therefore inhibiting cancer growth. Human and canine melanoma cells were used, and serial dilutions of the standard solution of *E. tirucalli* latex were tested. We verified that DETL was effective in reducing the viability of both human and canine melanoma cells. To our knowledge, this is the first study that investigates DETL effects on canine cells.

The significantly decreased cell viability in human melanoma cells was seen mostly at 48 and 72 hours after treatment in human melanoma cells. The characteristic U-shaped curve of cell viability, seen more prominently in human melanoma cells, may represent a hormesis effect. Hormesis effect is a biphasic dose response of a cell or organism to an environmental agent or substance characterized by low dose stimulation or beneficial effect on the cells and a high-dose inhibitory or toxic effect [22]. The lower doses produce an adaptive compensatory response to an initial disruption in homeostasis, leading to a cellular stress response and the consequent activation of stress resistance genes, growth factors, energy metabolism, heat shock proteins, and antioxidant enzymes that outweigh the toxicity [23]. Multiple studies have shown this effect [24–27]. It is important to state that low doses of chemicals reach tumor cells through circulation, and therefore our results may represent a possibility that DETL may act in tumor cells in vivo as well.

Canine melanoma cells had a significantly lower viability upon treatment with dilutions of 1/32 to 1/512 at 24 hours, 1/4 to 1/256 at 48 hours, and for all dilutions at 72 hours. DETL also determined a hormesis effect for canine melanoma cells. In another study, the cell viability of MCF-7 and Melan-A cell lines treated with 1% and 10% of high dilutions of latex for 24 h was assessed by MTT [21]. The authors verified that *Euphorbia tirucalli* 15 CH induced an increase in MCF-7 viability and concluded that high dilutions of *E. tirucalli* latex may interfere with the cell metabolism [15]. Regarding the mechanism of action by which cytotoxic activity occurs, it is likely that the antineoplastic activity of *E. tirucalli* latex may be the consequence of the synergistic activity of some active ingredients, such as esters of ingenol, as well as the group of lanosta-8,24-dien-3-ol molecules, defined as euphol, tirucallol, lanosterol, and derivatives, which act as inhibitors of protein kinases [28], with multitarget activity.

Recently, our group reported that DETL not only reduced the cell viability in vitro but was also effective in inhibiting metastasis of murine B16/F10 melanoma cells [29].

We must clarify that the composition of *E. tirucalli* latex may show probable geographic variations in its composition and antiproliferative activity [12]. The results obtained here refer to *E. tirucalli* grown in São Paulo city, São Paulo, Brazil. Therefore, our study does not validate the efficacy and toxicity of *E. tirucalli* latex from plants cultivated elsewhere.

The goal of any scientific study is to make discoveries that can solve intriguing problems. The success of an antineoplastic approach must take into account the efficacy and toxicity of the new therapy. Balancing these two is challenging and depends on a large number of factors [30]. If the compound shows efficacy at low doses, there is a lower chance of it to be toxic. Thus, we consider that the DETL has the potential to be used for antineoplastic therapy. Since dogs are affected by many types of tumors and must receive adequate treatment with conventional or alternative drugs, at low cost, DETL can be considered a potential new treatment for oral melanoma in this species. However, further studies are necessary to understand its toxicity in normal cells at the proposed dilutions, the components of *E. tirucalli* latex that are acting synergistically, and their mechanisms of action on cancer cells, and the balance between toxicity and therapeutic effects in vivo.

## Data Availability

The data used to support this study are available from the corresponding author on request.
